# Association between total and leisure time physical activity and risk of myocardial infarction and stroke – a Swedish cohort study

**DOI:** 10.1186/s12889-022-12923-5

**Published:** 2022-03-18

**Authors:** Madeleine Hummel, Essi Hantikainen, Hans-Olov Adami, Weimin Ye, Rino Bellocco, Stephanie Erika Bonn, Ylva Trolle Lagerros

**Affiliations:** 1 Clinical Epidemiology Division, Department of Medicine Solna, Karolinska Institutet, Eugeniahemmet T2:02, 171 76 Stockholm, Sweden; 2 Institute for Biomedicine, Affiliated to the University of Lübeck, Eurac Research, Bozen, Italy; 3 Department of Medical Epidemiology and Biostatistics, Karolinska Institutet, Stockholm, Sweden; 4 Clinical Effectiveness Group, Institute of Health and Society, University of Oslo, Oslo, Norway; 5 Department of Epidemiology and Health Statistics & Key Laboratory of Ministry of Education for Gastrointestinal Cancer, Fujian Medical University, Fuzhou, China; 6 Department of Statistics and Quantitative Methods, University of Milano-Bicocca, Milan, Italy; 7 Center for Obesity, Academic Specialist Center, Stockholm Health Services, Stockholm, Sweden

**Keywords:** Epidemiology, Exercise, Myocardial infarction, Risk factors, Stroke

## Abstract

**Background:**

Physical inactivity is a known risk factor for cardiovascular disease, but it is unclear if total and leisure time activity have different impact on the risk of myocardial infarction and stroke. In this cohort, we aimed to investigate the associations between both total and leisure time physical activity in detail, and the risks of myocardial infarction and stroke, both overall and for men and women separately.

**Methods:**

We assessed the association between total and leisure time physical activity on the risk of myocardial infarction and stroke in a cohort of 31,580 men and women through record linkages from 1997–2016. We used Cox proportional hazards regression models to estimate hazard ratios (HR) with 95% confidence intervals (CI) based on detailed self-reported physical activity. In the adjusted analyses, we included age, sex, body mass index, level of education, cigarette smoking, alcohol consumption, diabetes, lipid disturbance and hypertension as potential confounders.

**Results:**

We identified 1,621 incident cases of myocardial infarction and 1,879 of stroke. Among men, there was an inverse association between leisure time activity and myocardial infarction in the third tertile compared to the first (HR: 0.78; 95% CI: 0.62–0.98; *p* for trend = 0.03). We also found an inverse association between leisure time activity and stroke in the third tertile compared to the first (HR: 0.78; 95% CI: 0.61–0.99; *p* for trend = 0.04), while the corresponding HR for stroke among women was 0.91; 95% CI: 0.74–1.13. We found no significant association between total physical activity and MI (HR: 1.12; 95% CI: 0.93–1.34) or stroke (HR: 1.14 95% CI: 0.94–1.39) comparing the highest to the lowest tertile in men. Women in the highest tertile of total physical activity had a 22% lower risk of myocardial infarction compared to the lowest tertile (HR: 0.78; 95% CI: 0.63–0.97; *p* for trend = 0.02) and an 8% (95% CI: 0.87–0.98) reduced risk of myocardial infarction with each 1 METh/day increase of leisure time physical activity.

**Conclusion:**

Total physical activity was inversely associated with the risk of myocardial infarction in women, while leisure time physical activity was inversely associated with the risk of myocardial infarction and stroke in men.

**Supplementary Information:**

The online version contains supplementary material available at 10.1186/s12889-022-12923-5.

## Introduction

Physical inactivity is an important risk factor for cardiovascular disease (CVD), the leading cause of death worldwide [1] with an estimated 17.9 million deaths in 2016, of which 85% were due to stroke or myocardial infarction (MI) [2]. According to the Swedish Board of Health and Welfare, CVD death is the leading cause of death for both men and women in Sweden. It accounted for 31% of all deaths in 2019 [3]. In the past 20–30 years, reduced prevalence of risk factors, primarily treatment of hypertension and blood lipid disturbances in addition to smoking control, has highly contributed to a reduction in CVD mortality. However, this decline is partly counterbalanced by a lifestyle with increasing amounts of sedentary time [4, 5]. Only 7% of middle-aged Swedish men and women meet the Swedish national guidelines for physical activity recommendations [6].

Epidemiological studies have shown an inverse association between physical activity and CVD [7–9]. Physical activity has beneficial effects on a number of CVD risk factors such as diabetes type 2, and it improves artery function, as well as reduces and prevents atherogenic markers. Most studies have indicated that a small increase in physical activity from an inactive lifestyle provides the greatest gain in CVD health in both men and women [10]. Others suggest that physical activity affects the risk of CVD differently in men and women, favoring women [11, 12]. A previous meta-analysis found high levels of leisure time physical activity to be beneficial, while a high level of occupational activity was associated with an increased risk of CVD [13]. It could be hypothesized that more men than women have occupations with physically strenuous work tasks, such as bending and lifting without rest, which may be detrimental to health. Yet today many work-related activities are done sitting. Nevertheless, self-reported sedentary time has been shown to often be underestimated, while total physical activity has been shown to often be overestimated, compared to objective methods [14].

However, many studies assess only leisure time physical activity, which may have different impact on the risk of MI and stroke than total physical activity. To fill these knowledge gaps in a large prospective study with long follow-up time, we assessed both total and leisure time physical activity in detail, measured in METh/day, and the risk of MI and stroke in both men and women.

## Methods

### Study design, setting and participants

In September 1997, the Swedish National March, a fund-raising event organized by the Swedish Cancer Society, took place in almost 3,600 Swedish cities and villages. A general population cohort, the Swedish National March Cohort (SNMC), was established of people who took part in the event as described in detail previously [15]. All participants were asked to fill out a 36-page questionnaire, in which they provided information on background data and lifestyle factors with detailed information on physical activity. In total, 43,880 participants completed the questionnaire. They provided their national registration number, a unique personal identifier assigned to all Swedish residents at birth or immigration [16]. Through this, it was possible to link the cohort to different well-validated, national registers to identify MI and stroke, with virtually complete follow-up.

The study was approved by the Regional Ethical Review Board at Karolinska Institutet, Stockholm, Sweden (Dnr: 97–205 and 2017/796–31), and it was reported according to the STROBE Statement for reporting Observational Studies.

We excluded individuals who had died (*n* = 8) or emigrated (*n* = 41) before the start of the follow-up, who were below the age of 18 years at time of enrollment (*n* = 1,740), or had any history of cardiovascular disease (*n* = 4,733) according to the International Classifications of Diseases (ICD) versions 7 to 10 through linkage with the National Inpatient and Outpatient Register (ICD-7: 400–468; ICD-8: 330–334; ICD-9: 390–459; ICD-10: I00-I99). We further excluded those who had ever been diagnosed with cancer, except non-melanoma skin cancer (*n* = 2,682) through linkage with the National Cancer Register (ICD-coding for non-melanoma skin cancer: ICD-7: 191). Finally, we excluded individuals who had missing answers on questions regarding total physical activity (*n* = 3,674), or leisure time physical activity (*n* = 10,843), leaving us a final cohort of 31,580 and 24,211 subjects for the analyses on total physical activity and leisure time physical activity, respectively.

### Baseline measures

All baseline information was self-reported in the questionnaire, including smoking habits (never, former, current), alcohol consumption (≤ 3 times/month, 1–6 times/week, > 1 time/day), and educational level (< 13 years or ≥ 13 years). Information on prevalent hypertension, diabetes, and lipid disturbance was defined as having ever been treated by a physician for any of these conditions (yes or no). Height, weight, and waist circumference were measured by the participants themselves at baseline and reported in the questionnaire. Waist circumference was instructed to be measured at the umbilicus with a pictured instruction. Body mass index (BMI) was calculated by dividing reported weight (kg) by the squared height (m^2^) and categorized as normal weight (< 25 kg/m^2^), overweight (25–29.9 kg/m^2^), or obese (≥ 30 kg/m^2^), according to the standard classification of the World Health Organization. Waist circumference was categorized as central obesity (≥ 80 cm in women and ≥ 94 cm in men) and severe central obesity (≥ 88 cm in women and ≥ 102 cm in men), according to the International Diabetes Federation [17] and the World Health Organization [18].

### Exposures

Total physical activity was assessed by the question “How physically active are you on an ordinary weekday?”. Participants were asked to report the time spent at each of nine physical activity intensity levels, illustrated by common activities from sleeping to more strenuous activities e.g. shoveling snow by hand. The total time reported should sum up to 24 h. Each activity intensity level was assigned a MET value; 0.9 METs for the lowest activity to 1, 1.5, 2, 3, 4, 5, 6, and 8 METs for the most intense activity level. Total physical activity per day was estimated by summarizing all levels into total Metabolic Energy Turnover (MET)-h/day. A MET-hour is defined as the ratio of work metabolic rate to a standard resting metabolic rate of energy expenditure of 1 kcal per kg body weight per hour, which corresponds to the energy used for one hour of quiet sitting. One MET is considered the reference metabolic rate, and other activities are expressed as multiples of one MET. The higher the MET, the more intense the activity and the higher the energy cost [19, 20]. The questionnaire has previously been validated giving a Pearson correlation coefficient of 0.73 when compared with the mean of three 24 h recalls [21].

Leisure time physical activity was assessed in a separate section of the questionnaire by asking the participant "How much time per week, on average, during the last 12 months have you devoted to sports/exercise/athletics/outdoor life?". Participants were asked to report the average number of hours per week dedicated to such activities during summer and winter, respectively. The activities were divided into three levels: light physical activity corresponding to 3 METs (e.g. walking); strenuous physical activity corresponding to 6 METs (e.g. speedy walking, jogging, or swimming); and hard physical activity corresponding to 10 METs (e.g. vigorous exercise or competitive training). The number of hours were then multiplied by the corresponding MET-value for each intensity level and summed up to estimate total leisure time physical activity in MET-h/week. The question about leisure time activity is similar to the Godin Leisure-Time Exercise Questionnaire [22], which has been extensively validated [23, 24].

### Follow-up and outcome

Participants were followed from October 1, 1997 to the end of the follow-up on December 31, 2016, or to the date of emigration, death, or a first MI or stroke, whichever came first. Non-fatal events were ascertained through linkage to the Swedish National Inpatient and Outpatient Register, which covers all in-patient hospital discharge and outpatient (specialist care, since 2001) diagnoses, using the provided national registration numbers. Fatal events were ascertained in the Swedish Cause of Death Register. Information on emigration was obtained from the Swedish Population Register. We identified incident cases using the International Classifications of Diseases (ICD) codes; 410 (ICD-9), and I21 (ICD-10) for myocardial infarction; 430, 433, 434, 436 (ICD-9), and I60, I61, I63.0-I63.5, I63.8-I63.9, I64 (ICD-10) for stroke, in the National Inpatient Register and the Cause of Death Register. The validity of the diagnoses recorded in the National Inpatient Register has previously been evaluated. The positive predictive value (PPV) for MI varies between 98–100% and for stroke between 68.5%-98.6%; the estimated sensitivity between 77.0–91.5% for MI and 84.2–95.0% for stroke [25].

### Statistical methods

Participants’ baseline characteristics are reported as means (standard deviation) for continuous variables and percent for categorical variables. The distribution of total physical activity and leisure time physical activity were categorized into sex-specific tertiles.

When reporting total physical activity, there was a tendency to under-report number of hours in a day. When the sum of time did not make it up to 24 h, we assumed that the missing time had been sleep or rest, thus, we multiplied missing hours with the basal metabolic rate. When the sum of the reported data was more than 24 h, we multiplied each level-specific value by 24 divided by the reported total number of hours, thus assuming that the misrepresentation of time was constant per time unit and independent of the physical intensity level.

We fitted Cox proportional hazards regression models to estimate hazard ratios (HRs) with corresponding 95% confidence intervals (CIs) for MI and stroke incidence at different levels of total physical activity and leisure time physical activity, with the lowest levels of each domain used as the reference category. In addition, we also fitted models with total physical activity and leisure time physical activity as continuous variables. Age was used as the underlying time scale.

We selected potential confounders based on known risk factors of MI and stroke, and used the web-based application DAGitty to draw directed acyclic graphs (Supplementary Fig. 1) [26]. As potential confounders, we included age, sex, BMI (normal < 25, overweight 25–29.9, obese ≥ 30), level of education (< 13 or ≥ 13 years), cigarette smoking (never, former, current), and alcohol consumption (all types of alcoholic beverage: never, low: ≤ 3 times/month, medium: 1–6 time/week, high: ≥ 1 time/day), in our statistical models. Diabetes (self-reported, yes or no), lipid disturbance (self-reported, yes or no), and hypertension (self-reported, yes or no) may be on the causal pathway between physical activity and MI and stroke, or they may be confounders, as these conditions might actually affect personal physical activity. Therefore, we first fitted models without adjustment for diabetes, lipid disturbance and hypertension and second with further adjustment for these factors. The main analyses were repeated for males and females separately.

Ties were handled using the Breslow method [27] and the proportional hazards assumption was tested by using Schoenfeld’s residuals. If the assumption was violated, stratified models were fitted. To further examine linear trends, a new categorical variable based on the median of each physical activity tertile was created and implemented as a continuous variable in the models. In addition, the dose–response relationship was investigated using restricted cubic splines with three knots placed at the 10th, 50th and 90th percentile of the distribution of total physical activity and leisure time physical activity (Supplementary Fig. 2a-l) [28].

We further investigated the role of potential effect modifiers, i.e. sex, BMI, smoking, alcohol consumption and age at baseline, on the relationship between total and leisure time physical activity with MI and stroke on the multiplicative scale. To do so, we included the cross-product interaction term with the variables of interest in the main models and used the likelihood ratio test to compare nested models. The potential effect modifiers were implemented as follows in the models: sex (female, male), BMI (≤ 25 kg/m^2^, > 25 kg/m^2^), smoking (never and former, current), alcohol consumption (low, medium, high), and age at baseline (< 60 years, ≥ 60 years).

We further conducted a sensitivity analysis by excluding cases of MI or stroke occurring during the first two years of follow-up and the corresponding person-years to reduce possible impact of reversed causality. Because physical activity was assessed only at baseline which precluded assessment of changes in physical activity, we further investigated whether the association between each exposure and outcome variable was affected by restricting the follow-up time to 10 years from baseline. As another sensitivity analysis, we repeated the main analyses after categorizing the exposure variables into 9 to investigate the effect of extreme high vs. extreme low physical activity. Moreover, because 8,986 participants had missing information on waist circumference, we adjusted for waist circumference (cm) in a last sensitivity analysis.

The proportions of missing data within the two exposure variables were 10.4% for total physical activity and 30.8% for leisure time physical activity. Missing values of the covariates were 4.4% for BMI, 1.1% for level of education, 7.8% for smoking, 0.01% for alcohol consumption, 3.9% for diabetes, 4.3% for lipid disturbance, and 3.3% for hypertension. Under the assumption of data missing at random [29, 30], we conducted a multiple imputation analysis and fitted multiple imputation models based on chained equations using Rubin’s rules [31] to estimate pooled effect estimates and standard errors. Analyses were performed using Stata 15.1 (Stata Corporation, College Station, TX, USA).

## Results

Demographic characteristics at baseline by level of total physical activity among men and women are shown in Table 1. The cohort consisted of 31,580 participants, of which 10,725 (34%) were men and 20,855 (66%) were women. During the mean follow-up period of 17.9 years, there were 1,621 incident cases of MI and 1,879 of stroke. At baseline, participants in the highest tertile of total physical activity were more likely to be younger, less educated, to have a lower BMI, and a lower waist circumference than participants in lower tertiles of total physical activity.

**Table 1:** Characteristics of study participants by categories of levels of total physical activity

**Sex-specific tertiles of total physical activity (METh/day)**	Low	Medium	High	*P* value
*Male*	21.8–34.5	> 34.5–45.8	> 45.8–140.7	
*Female*	22.0–31.7	> 31.7–38.2	> 38.2–128.9	
***Overall population***				
Number of participants	10,527	10,527	10,527	
Age (years), mean (SD)	50.7 (16.0)	48.9 (15.0)	47.6 (16.2)	0.00
BMI (kg/m^2^), mean (SD)	24.8 (3.7)	24.4 (3.4)	24.2 (3.3)	0.00
Normal, < 25 kg/m^2^ (%)	57.5	62.4	65.4	0.00
Overweight, 25–29.9 kg/m^2^ (%)	33.7	31.6	29.3	
Obese, ≥ 30 kg/m^2^ (%)	8.8	6.0	5.4	
Waist circumference (cm), mean (SD)	85.7 (12.0)	84.0 (11.6)	82.8 (11.4)	0.00
Normal < 94 cm	75.4	80.1	82.5	
Central obesity, ≥ 94 cm—< 102 cm (%)	15.3	13.1	12.0	
Severe central obesity, ≥ 102 cm (%)	9.4	6.8	5.5	
Smoking (%)				0.00
Never	63.8	64.5	66.7	
Former	27.7	27.8	25.4	
Current	8.6	7.7	7.9	
Alcohol consumption (%)				0.00
≤ 3 times/month	47.1	45.6	46.3	
1–6 times/week	50.7	52.7	52.0	
> 1 time/day	2.3	1.8	1.7	
Level of education, ≥ 13 years (%)	31.7	34.6	25.3	0.00
Diabetes, yes (%)	2.1	1.8	1.9	0.38
Lipid disturbance, yes (%)	2.8	2.0	2.2	0.00
Hypertension, yes (%)	11.4	8.9	8.4	0.00
Daily aspirin use, yes (%)	20.0	18.5	17.1	0.00
Leisure time physical activity, METh/day (%)				
Low, ≤ 1.6 METh/day	49.3	30.5	21.6	0.00
Medium, > 1,6–3.6 METh/day	34.9	36.5	28.8	
High, > 3.6 METh/day	15.8	33.1	49.6	
Sitting time, ≥ 6 h/day (%)	41.3	47.0	17.3	0.00
***Male***				
Number of participants	3,575	3,575	3,575	
Age (years), mean (SD)	50.9 (16.6)	50.9 (16.2)	48.9 (18.1)	0.00
BMI (kg/m^2^), mean (SD)	25.1 (3.3)	24.9 (2.8)	24.8 (2.9)	0.00
Normal, < 25 kg/m^2^ (%)	52.2	55.8	57.3	
Overweight, 25–29.9 kg/m^2^ (%)	40.8	39.7	37.9	
Obese, ≥ 30 kg/m^2^ (%)	7.1	4.5	4.7	
Waist circumference (cm), mean (SD)	94.0 (10.9)	92.3 (10.1)	91.4 (10.8)	0.00
Normal < 94 cm	48.6	56.6	58.6	
Central obesity, ≥ 94 cm—< 102 cm (%)	30.4	28.1	27.6	
Severe central obesity, ≥ 102 cm (%)	21.0	15.3	13.7	
Smoking (%)				0.00
Never	60.8	63.0	65.7	
Former	31.2	30.7	28.0	
Current	8.0	6.4	6.3	
Alcohol consumption (%)				0.21
≤ 3 times/month	29.5	31.0	30.9	
1–6 times/week	66.7	65.5	66.2	
> 1 time/day	3.8	3.5	3.0	
Level of education, ≥ 13 years (%)	33.5	30.4	14.5	0.00
Diabetes, yes (%)	2.8	2.5	2.5	0.71
Lipid disturbance, yes (%)	2.7	2.3	2.4	0.59
Hypertension, yes (%)	10.6	9.4	9.2	0.10
Daily aspirin use, yes (%)	16.3	15.3	14.4	0.09
Leisure time physical activity, METh/day (%)				
Low, ≤ 1.6 METh/day	49.3	27.7	25.3	0.00
Medium, > 1,6–3.6 METh/day	35.6	35.9	28.2	
High, > 3.6 METh/day	15.1	36.4	46.5	
Sitting time, ≥ 6 h/day (%)	49.8	41.5	9.9	0.00
***Female***				
Number of participants	6,952	6,952	6,951	
Age (years), mean (SD)	50.5 (15.7)	47.9 (14.2)	46.9 (15.1)	0.00
BMI (kg/m^2^), mean (SD)	24.7 (3.9)	24.2 (3.6)	23.9 (3.4)	0.00
Normal, < 25 kg/m^2^ (%)	60.3	65.8	69.5	
Overweight, 25–29.9 kg/m^2^ (%)	30.0	27.4	24.8	
Obese, ≥ 30 kg/m^2^ (%)	9.7	6.8	5.7	
Waist circumference (cm), mean (SD)	81.9 (10.5)	80.3 (10.2)	79.1 (9.6)	0.00
Normal < 80 cm (%)	43.9	51.4	56.7	
Central obesity, ≥ 80 cm – < 88 cm (%)	30.3	29.0	26.4	
Severe central obesity, ≥ 88 cm (%)	25.8	19.6	16.9	
Smoking (%)				0.03
Never	65.2	65.2	67.1	
Former	25.9	26.4	24.1	
Current	8.9	8.4	8.8	
Alcohol consumption (%)				0.00
≤ 3 times/month	56.2	53.1	54.2	
1–6 times/week	42.4	46.0	44.8	
> 1 time/day	1.5	0.92	1.0	
Level of education, ≥ 13 years (%)	30.8	36.8	30.8	0.00
Diabetes, yes (%)	1.7	1.5	1.6	0.54
Lipid disturbance, yes (%)	2.8	1.8	2.1	0.00
Hypertension, yes (%)	11.9	8.6	8.0	0.00
Daily aspirin use, yes (%)	21.9	20.1	18.4	0.00
Use of contraceptive pills, yes (%)	65.1	71.0	68.6	0.00
Use of hormone replacement therapy, yes (%)	29.8	25.5	22.9	0.00
Leisure time physical activity, METh/day (%)				0.00
Low, ≤ 1.5 METh/day	49.3	31.8	19.8	
Medium, > 1,5–3,1 METh/day	34.5	36.7	29.1	
High > 3,1 METh/day	16.2	31.5	51.1	
Sitting time, ≥ 6 h/day (%)	37.0	49.8	21.2	0.00

In Supplementary Table 1, we additionally present the distribution of METh across physical activity categories during a weekday for men and women. Men tended to have higher METh from activities with a MET ranging from 3–8, such as mowing the lawn, shoveling snow and more strenuous activities, whereas women tended to have higher METh in activities with a MET ranging from 1.5–2 (office work, household chores).

### Total physical activity

Table 2 shows results of total physical activity and risk of MI. We found no association between total physical activity and MI among all participants. When men and women were analyzed separately, no association between total physical activity and MI was found among men. In women, we found an inverse association between total physical activity and MI, with the lowest HR in the highest tertile (HR: 0.78; 95% CI: 0.63–0.97; *p* for trend = 0.02), compared to the lowest. Similarly, when implementing the exposure variable as a continuous variable in the model, we found a 1% lower risk of MI in women (95% CI: 0.98–0.99) with each 1 METh/d increase in total physical activity in the adjusted model.

**Table 2:** Incidence rates and hazard ratios of myocardial infarction for total physical activity

**Sex-specific tertiles of total physical activity (METh/day)**	Continuous (per 1METh/day increase)	Low	Medium	High	*P* for linear trend
*Male*		21.8–34.5	> 34.5–45.8	> 45.8–140.7	
*Female*		22.0–31.7	> 31.7–38.2	> 38.2–128.9	
*Total*					
Myocardial infarction					
Number of events		599	523	499	
Person-years (py)		186,508	189,339	189,636	
Event rate per 100,000 py^a^		321.2	276.2	263.1	
HR (95% CI)^a^	1.00 (0.99–1.00)	1.00 (reference)	0.98 (0.87–1.10)	0.96 (0.85–1.08)	0.51
HR (95% CI)^b^	1.00 (0.99–1.00)	1.00 (reference)	1.03 (0.91–1.17)	0.95 (0.83–1.08)	0.34
HR (95% CI)^c^	1.00 (0.99–1.00)	1.00 (reference)	1.04 (0.91–1.18)	0.96 (0.84–1.10)	0.48
*Male*					
Myocardial infarction					
Number of events		302	297	315	
Person-years		61,118	61,686	61,840	
Event rate per 100,000 py^a^		494.1	481.5	509.4	
HR (95% CI)^a^	1.00 (0.99–1.00)	1.00 (reference)	0.97 (0.83–1.14)	1.08 (0.92–1.26)	0.26
HR (95% CI)^b^	1.00 (0.99–1.00)	1.00 (reference)	0.99 (0.84–1.18)	1.06 (0.89–1.26)	0.48
HR (95% CI)^c^	1.00 (0.99–1.00)	1.00 (reference)	1.05 (0.88–1.25)	1.12 (0.93–1.34)	0.24
*Female*					
Myocardial infarction					
Number of events		297	226	184	
Person-years		125,391	127,653	127,797	
Event rate per 100,000 py^a^		236.9	177.0	144.0	
HR (95% CI)^a^	1.00 (0.99–1.00)	1.00 (reference)	1.01 (0.85–1.20)	0.82 (0.68–0.98)	0.02
HR (95% CI)^b^	1.00 (0.99–1.00)	1.00 (reference)	1.11 (0.92–1.34)	0.82 (0.67–1.00)	0.03
HR (95% CI)^c^	0.99 (0.98–0.99)	1.00 (reference)	1.07 (0.88–1.31)	0.78 (0.63–0.97)	0.02

*HR* Hazard ratio, *CI* Confidence interval, *PY* Person-years

^a^Adjusted for age and sex at enrollment

^b^Adjusted for age, cigarette smoking, alcohol consumption, level of education, and body mass index

^c^Adjusted for age, cigarette smoking, alcohol consumption, level of education, body mass index, hypertension, diabetes, and lipid disturbance

We found no association between total physical activity and stroke in men or women, nor when ischemic and hemorrhagic stroke were analyzed separately (Table 3).

**Table 3:** Incidence rates and hazard ratios of stroke for total physical activity

**Sex-specific tertiles of total physical activity (METh/day)**	Continuous (per1METh/day increase)	Low	Medium	High	*P* value for trend
*Male*		21.8–34.5	> 34.5–45.8	> 45.8–140.7	
*Female*		22.0–31.7	> 31.7–38.2	> 38.2–128.9	
*Total*					
Stroke, ischemic and hemorrhagic					
Number of events		680	621	578	
Person-years (py)		186,105	188,926	189,091	
Event rate per 100,000 py^a^		365.3	328.7	305.7	
HR (95% CI)^a^	1.00 (0.99–1.00)	1.00 (reference)	1.05 (0.94–1.17)	1.00 (0.89–1.12)	0.84
HR (95% CI)^b^	1.00 (0.99–1.00)	1.00 (reference)	1.04 (0.92–1.17)	0.99 (0.88–1.12)	0.76
HR (95% CI)^c^	1.00 (0.99–1.00)	1.00 (reference)	1.09 (0.96–1.23)	1.04 (0.92–1.19)	0.66
Stroke, ischemic					
Number of events		468	397	408	
Person-years		186,105	188,926	189,091	
Event rate per 100,000 py^a^		251.5	210.1	215.8	
HR (95% CI)^a^	1.00 (0.99–1.00)	1.00 (reference)	0.99 (0.86–1.13)	1.03 (0.90–1.18)	0.58
HR (95% CI)^b^	1.00 (0.99–1.00)	1.00 (reference)	0.97 (0.84–1.12)	1.02 (0.88–1.18)	0.73
HR (95% CI)^c^	1.00 (0.99–1.00)	1.00 (reference)	1.02 (0.87–1.19)	1.07 (0.92–1.25)	0.34
Stroke, hemorrhagic					
Number of events		114	114	84	
Person-years		186,105	188,926	189,091	
Event rate per 100,000 py^a^		61.3	60.3	44.4	
HR (95% CI)^a^	1.00 (0.99–1.00)	1.00 (reference)	1.11 (0.85–1.44)	0.83 (0.63–1.11)	0.13
HR (95% CI)^b^	1.00 (0.99–1.00)	1.00 (reference)	1.08 (0.81–1.42)	0.85 (0.63–1.14)	0.20
HR (95% CI)^c^	1.00 (0.99–1.00)	1.00 (reference)	1.15 (0.86–1.54)	0.93 (0.68–1.27)	0.48
*Male*					
Stroke, ischemic and hemorrhagic					
Number of events		254	289	285	
Person-years		61,492	61,944	62,055	
Event rate per 100,000 py^a^		413.1	466.6	459.3	
HR (95% CI)^a^	1.00 (0.99–1.00)	1.00 (reference)	1.13 (0.95–1.34)	1.15 (0.97–1.37)	0.15
HR (95% CI)^b^	1.00 (0.99–1.00)	1.00 (reference)	1.10 (0.92–1.32)	1.12 (0.93–1.35)	0.29
HR (95% CI)^c^	1.00 (0.99–1.00)	1.00 (reference)	1.13 (0.94–1.37)	1.14 (0.94–1.39)	0.25
Stroke, ischemic					
Number of events		178	194	205	
Person-years		61,492	61,944	62,055	
Event rate per 100,000 py^a^		289.5	313.2	330.4	
HR (95% CI)^a^	1.00 (0.99–1.00)	1.00 (reference)	1.08 (0.88–1.33)	1.18 (0.97–1.48)	0.10
HR (95% CI)^b^	1.00 (0.99–1.00)	1.00 (reference)	1.07 (0.86–1.34)	1.18 (0.94–1.47)	0.16
HR (95% CI)^c^	1.00 (0.99–1.00)	1.00 (reference)	1.13 (0.90–1.42)	1.21 (0.96–1.52)	0.14
Stroke, hemorrhagic					
Number of events		45	44	37	
Person-years		61,492	61,944	62,055	
Event rate per 100,000 py^a^		73.2	71.0	9.6	
HR (95% CI)^a^	1.00 (0.98–1.00)	1.00 (reference)	0.97 (0.64–1.47)	0.85 (0.55–1.31)	0.44
HR (95% CI)^b^	0.99 (0.98–1.00)	1.00 (reference)	0.82 (0.52–1.27)	0.78 (0.49–1.23)	0.34
HR (95% CI)^c^	0.99 (0.98–1.00)	1.00 (reference)	0.82 (0.51–1.30)	0.83 (0.51–1.33)	0.51
*Female*					
Stroke, ischemic and hemorrhagic					
Number of events		426	332	293	
Person-years		124,613	126,982	127,036	
Event rate per 100,000 py^a^		341.9	261.5	230.6	
HR (95% CI)^a^	1.00 (0.99–1.00)	1.00 (reference)	1.00 (0.87–1.16)	0.89 (0.77–1.04)	0.12
HR (95% CI)^b^	1.00 (0.99–1.00)	1.00 (reference)	1.00 (0.86–1.18)	0.91 (0.77–1.07)	0.21
HR (95% CI)^c^	1.00 (0.99–1.00)	1.00 (reference)	1.07 (0.91–1.26)	0.98 (0.83–1.17)	0.74
Stroke, ischemic					
Number of events		290	203	203	
Person-years		124,612	126,982	127,036	
Event rate per 100,000 py^a^		232.7	159.9	159.8	
HR (95% CI)^a^	1.00 (0.99–1.00)	1.00 (reference)	0.92 (0.77–1.10)	0.93 (0.77–1.11)	0.46
HR (95% CI)^b^	1.00 (0.99–1.00)	1.00 (reference)	0.91 (0.75–1.11)	0.92 (0.76–1.12)	0.45
HR (95% CI)^c^	1.00 (0.99–1.00)	1.00 (reference)	0.96 (078–1.17)	1.00 (0.82–1.23)	0.94
Stroke, hemorrhagic					
Number of events		69	70	47	
Person-years		124,613	126,982	127,036	
Event rate per 100,000 py^a^		55.4	55.1	37.0	
HR (95% CI)^a^	0.99 (0.98–1.00)	1.00 (reference)	1.21 (0.86–1.69)	0.82 (0.56–1.19)	0.19
HR (95% CI)^b^	0.99 (0.97–1.00)	1.00 (reference)	1.30 (0.91–1.86)	0.90 (0.61–1.34)	0.40
HR (95% CI)^c^	0.99 (0.98–1.00)	1.00 (reference)	1.45 (0.99–2.11)	1.01 (0.67–1.53)	0.73

*HR* Hazard ratio, *CI* Confidence interval, *PY* Person-years

^a^Adjusted for age and sex at enrollment

^b^Adjusted for age, cigarette smoking, alcohol consumption, level of education, and body mass index

^c^Adjusted for age, cigarette smoking, alcohol consumption, level of education, body mass index, hypertension, diabetes, and lipid disturbance

### Leisure time physical activity

Findings for leisure time physical activity and the risk of MI are presented in Table 4. We found an inverse association between leisure time physical activity and MI among all participants, with a 21% lower risk of MI in the highest tertile compared to the lowest tertile after adjusting for potential confounders, (HR: 0.79; 95% CI: 0.66–0.94; *p* for trend =  < 0.01). Among both men and women, the reduced risk in the highest tertile compared to the lowest tertile after adjusting for potential confounders was about 20%, with a statistically significant trend only among men. However, when investigating the exposure as a continuous variable the effect was only significant in women, showing an 8% (95% CI: 0.87–0.98) reduced risk of MI with each 1 METh/day increase from leisure time physical activity.

**Table 4:** Incidence rates and hazard ratios of myocardial infarction for leisure time physical activity

**Sex-specific tertiles of leisure** **time physical activity (METh/day)**	Continuous (per 1METh/day increase)	Low	Medium	High	*P* for linear trend
*Male*		0–1.6	> 1.6–3.6	> 3.6–16.3	
*Female*		0–1.5	> 1.5–3.1	> 3.1–16.3	
*Total*					
Myocardial infarction					
Number of events		428	404	250	
Person-years (py)		148,444	145,680	147,299	
Event rate per 100,000 py^a^		288.3	277.3	169.7	
HR (95% CI)^a^	0.96 (0.94–0.99)	1.00 (reference)	0.88 (0.77–1.01)	0.75 (0.64–0.87)	0.00
HR (95% CI)^b^	0.98 (0.95–1.01)	1.00 (reference)	0.92 (0.79–1.06)	0.81 (0.68–0.95)	0.01
HR (95% CI)^c^	0.97 (0.94–1.01)	1.00 (reference)	0.89 (0.76–1.03)	0.79 (0.66–0.94)	0.01
*Male*					
Myocardial infarction					
Number of events		234	239	143	
Person-years		47,753	46,096	47,342	
Event rate per 100,000 py^a^		490.0	518.5	302.1	
HR (95% CI)^a^	0.98 (0.95–1.01)	1.00 (reference)	0.92 (0.77–1.11)	0.76 (0.61–0.93)	0.01
HR (95% CI)^b^	0.99 (0.96–1.03)	1.00 (reference)	0.96 (0.79–1.17)	0.80 (0.64–1.00)	0.04
HR (95% CI)^c^	0.99 (0.96–1.03)	1.00 (reference)	0.96 (0.78–1.17)	0.78 (0.62–0.98)	0.03
*Female*					
Myocardial infarction					
Number of events		194	165	107	
Person-years		100,691	99,584	99,957	
Event rate per 100,000 py^a^		192.7	165.7	107.0	
HR (95% CI)^a^	0.92 (0.87–0.97)	1.00 (reference)	0.83 (0.67–1.02)	0.74 (0.58–0.93)	0.01
HR (95% CI)^b^	0.93 (0.88–0.99)	1.00 (reference)	0.86 (0.68–1.07)	0.82 (0.64–1.06)	0.14
HR (95% CI)^c^	0.92 (0.87–0.98)	1.00 (reference)	0.80 (0.63–1.01)	0.80 (0.61–1.04)	0.10

*HR* Hazard ratio, *CI* Confidence interval, *PY* Person-years

^a^Adjusted for age and sex at enrollment

^b^Adjusted for age, cigarette smoking, alcohol consumption, level of education, and body mass index

^c^Adjusted for age, cigarette smoking, alcohol consumption, level of education, body mass index, hypertension, diabetes, and lipid disturbance

An inverse borderline significant association was found between leisure time physical activity and stroke among all participants in the multivariable adjusted model (HR: 0.86; 95% CI: 0.73–1.00; *p* for trend = 0.06) (Table 5). When ischemic and hemorrhagic stroke were analyzed separately, an inverse association was found for ischemic stroke in the highest tertile compared to the lowest (HR: 0.81; 95% CI: 0.69–0.97; *p* for trend = 0.02), but this was not confirmed in the multivariable-adjusted models (*p* for trend = 0.31). No association was seen with hemorrhagic stroke when using the exposure variable categorized into tertiles. However, each 1 METh/day increase in leisure time physical activity was associated with a 7% (95% CI: 0.86–0.99) lower risk of hemorrhagic stroke among all participants.

**Table 5:** Incidence rates and hazard ratios of stroke for leisure time physical activity

**Sex-specific tertiles of leisure time physical activity (METh/day)**	Continuous (per 1METh/day increase)	Low	Medium	High	*P* for linear trend
*Male*		0–1.6	> 1.6–3.6	> 3.6–16.3	
*Female*		0–1.5	> 1.5–3.1	> 3.1–16.3	
*Total*					
Stroke, ischemic and hemorrhagic					
Number of events		490	461	307	
Person-years (py)		148,334	145,194	147,037	
Event rate per 100,000 py^a^		330.3	317.5	208.8	
HR (95% CI)^a^	0.96 (0.94–0.99)	1.00 (reference)	0.88 (0.77–1.00)	0.79 (0.69–0.91)	0.01
HR (95% CI)^b^	0.97 (0.94–0.99)	1.00 (reference)	0.92 (0.80–1.05)	0.83 (0.71–0.97)	0.02
HR (95% CI)^c^	0.97 (0.94–1.00)	1.00 (reference)	0.93 (0.81–1.07)	0.86 (0.73–1.00)	0.06
Stroke, ischemic					
Number of events		332	313	213	
Person-years		148,334	145,194	147,037	
Event rate per 100,000 py^a^		223.8	215.6	144.9	
HR (95% CI)^a^	0.97 (0.94–1.01)	1.00 (reference)	0.87 (0.75–1.02)	0.81 (0.69–0.97)	0.02
HR (95% CI)^b^	0.99 (0.95–1.02)	1.00 (reference)	0.93 (0.79–1.10)	0.87 (0.72–1.04)	0.14
HR (95% CI)^c^	0.99 (0.96–1.02)	1.00 (reference)	0.93 (0.78–1.10)	0.90 (0.74–1.09)	0.31
Stroke, hemorrhagic					
Number of events		96	75	58	
Person-years		148,334	145,194	147,037	
Event rate per 100,000 py^a^		64.7	51.7	39.4	
HR (95% CI)^a^	0.93 (0.87–0.99)	1.00 (reference)	0.74 (0.55–1.01)	0.75 (0.54–1.04)	0.09
HR (95% CI)^b^	0.93 (0.86–0.99)	1.00 (reference)	0.76 (0.55–1.04)	0.74 (0.53–1.05)	0.11
HR (95% CI)^c^	0.93 (0.86–0.99)	1.00 (reference)	0.76 (0.55–1.06)	0.75 (0.52–1.08)	0.14
*Male*					
Stroke, ischemic and hemorrhagic					
Number of events		203	221	127	
Person-years		48,129	46,193	47,516	
Event rate per 100,000 py^a^		421.8	478.4	267.3	
HR (95% CI)^a^	0.97 (0.93–1.00)	1.00 (reference)	0.96 (0.79–1.16)	0.75 (0.60–0.94)	0.01
HR (95% CI)^b^	0.97 (0.94–1.01)	1.00 (reference)	0.95 (0.77–1.16)	0.78 (0.61–0.98)	0.03
HR (95% CI)^c^	0.97 (0.93–1.01)	1.00 (reference)	0.94 (0.76–1.16)	0.78 (0.61–0.99)	0.04
Stroke, ischemic					
Number of events		147	146	95	
Person-years		48,129	46,193	47,516	
Event rate per 100,000 py^a^		305.4	316.1	199.9	
HR (95% CI)^a^	0.98 (0.94–1.03)	1.00 (reference)	0.87 (0.69–1.09)	0.77 (0.60–1.00)	0.06
HR (95% CI)^b^	0.99 (0.95–1.03)	1.00 (reference)	0.84 (0.66–1.08)	0.77 (0.58–1.01)	0.07
HR (95% CI)^c^	0.98 (0.94–1.03)	1.00 (reference)	0.82 (0.64–1.06)	0.75 (0.56–1.00)	0.06
Stroke, hemorrhagic					
Number of events		35	41	20	
Person-years		48,129	46,193	47,516	
Event rate per 100,000 py^a^		72.7	88.8	42.1	
HR (95% CI)^a^	0.93 (0.85–1.02)	1.00 (reference)	1.08 (0.69–1.70)	0.70 (0.40–1.21)	0.18
HR (95% CI)^b^	0.96 (0.87–1.05)	1.00 (reference)	1.14 (0.70–1.86)	0.83 (0.47–1.48)	0.49
HR (95% CI)^c^	0.96 (0.87–1.06)	1.00 (reference)	1.16 (0.69–1.93)	0.87 (0.48–1.58)	0.59
*Female*					
Stroke, ischemic and hemorrhagic					
Number of events		287	240	180	
Person-years		100,205	99,001	99,522	
Event rate per 100,000 py^a^		286.4	242.4	180.9	
HR (95% CI)^a^	0.95 (0.92–0.99)	1.00 (reference)	0.82 (0.69–0.97)	0.82 (0.68–0.99)	0.05
HR (95% CI)^b^	0.96 (0.92–1.00)	1.00 (reference)	0.89 (0.74–1.07)	0.88 (0.72–1.07)	0.23
HR (95% CI)^c^	0.97 (0.93–1.02)	1.00 (reference)	0.92 (0.76–1.11)	0.91 (0.74–1.13)	0.43
Stroke, ischemic					
Number of events		185	167	118	
Person-years		100,205	99,0001	99,522	
Event rate per 100,000 py^a^		184.6	168.7	118.6	
HR (95% CI)^a^	0.96 (0.92–1.01)	1.00 (reference)	0.88 (0.72–1.09)	0.85 (0.67–1.07)	0.17
HR (95% CI)^b^	0.98 (0.93–1.03)	1.00 (reference)	1.00 (0.80–1.25)	0.95 (0.74–1.23)	0.70
HR (95% CI)^c^	0.99 (0.94–1.05)	1.00 (reference)	1.02 (0.80–1.28)	1.03 (0.79–1.33)	0.84
Stroke, hemorrhagic					
Number of events		61	34	38	
Person-years		100,205	99,001	99,521	
Event rate per 100,000 py^a^		60.9	34.4	38.2	
HR (95% CI)^a^	0.92 (0.84–1.02)	1.00 (reference)	0.55 (0.36–0.83)	0.78 (0.52–1.18)	0.30
HR (95% CI)^b^	0.89 (0.80–0.99)	1.00 (reference)	0.53 (0.35–0.84)	0.69 (0.45–1.08)	0.14
HR (95% CI)^c^	0.89 (0.80–0.99)	1.00 (reference)	0.56 (0.36–0.88)	0.70 (0.44–1.09)	0.15

*HR* Hazard ratio, *CI* Confidence interval, *PY* Person-years

^a^Adjusted for age and sex at enrollment

^b^Adjusted for age, cigarette smoking, alcohol consumption, level of education, and body mass index

Further, we found an inverse association between leisure time physical activity and total stroke in men, with a 25% lower risk in the highest tertile compared to the lowest tertile, which remained significant after adjusting for potential confounders (HR: 0.78; 95% CI: 0.61–0.99; *p* for trend = 0.04). Among women, no significant association was found between leisure time physical activity and stroke overall, nor for ischemic and hemorrhagic stroke.

When we repeated the analyses after categorizing the exposure variable into deciles, we similarly found an inverse association between leisure time physical activity and the risk of MI in the fully adjusted model with a 41% lower risk in subjects in the ninth (HR: 0.59; 95% CI: 0.43–0.82) and a 22% lower risk in subjects in the highest decile (HR: 0.78: 95% CI: 0.56–1.10) compared to the lowest, with a significant trend (*p* for trend = 0.03). Similarly, there was an inverse association between leisure time physical activity and total stroke with a 36% lower risk for stroke in subjects in the seventh (HR: 0.64; 95% CI: 0.49–0.86), followed by a 30% lower risk for subjects in the highest decile compared to the lowest, with a significant trend (*p* for trend = 0.048). No association was found for total physical activity and MI or stroke (data not shown).

When further investigating the dose–response relationship we found no deviation from a linear association, for any of the physical activity domains with MI or stroke.

### Results from additional analyses

We found no statistical evidence for effect modification by BMI, smoking, alcohol consumption and age at baseline (all *p*-values for the LR-test comparing nested models > 0.05). Sex was an effect modifier of the relationship between physical activity and MI, as shown in Table 2 (*p*-value for the LR-test comparing nested models < 0.05). However, there was no significant evidence for effect modification by sex in the other models (*p*-values for the LR-test comparing nested models > 0.05).

In sensitivity analyses excluding stroke and MI cases occurring during the first two years of follow up, the effect of leisure time physical activity on the risk of total stroke was not statistically significant in the multivariable adjusted model in the overall population (*p* for trend = 0.07), nor in the subgroup of men (*p* for trend = 0.06). Estimates for the other models were not affected by this sensitivity analysis (data not shown).

After restricting the follow-up time to 10 years from baseline, our findings remained similar. However, they did not reach statistical significance in the subgroup analysis investigating the association between leisure time physical activity and ischemic stroke in men (*p* for trend = 0.10). When further adjusting our main models for waist circumference, our estimates stayed essentially the same (data not shown). Finally, when repeating our main models after using imputed missing data we obtained similar results (data not shown).

## Discussion

In this large prospective study, leisure time physical activity, including sports and outdoor life, were inversely associated with the risk of MI among all participants and the risk of MI and stroke in men. Total physical activity, including all physical activities on an ordinary day, summing up to 24 h, was inversely associated with the risk of MI in women.

Many studies have investigated CVD as a combined outcome [32, 33]. Not all have studied the effect on MI and stroke, nor in men and women, separately. In one cohort study [34], a high leisure time physical activity was associated with a 22% lower risk of CVD events among 2,352 men and women. In another study [35], lack of leisure time physical activity among men above the age of 45 was associated with a twofold increased risk of non-fatal acute MI. Furthermore, in a prospective study, a 28% reduced risk of stroke was found among 22,841 Finnish men with high compared to low leisure time physical activity [36], which is consistent with our findings of a 22% lower risk among men. Overall, our results for leisure time physical activity are in line with results from previous studies [34–37], showing a lower risk of MI and stroke with increasing activity level, although in our study this association was confined to men. Comparable with our findings, Finnish men with high leisure time physical activity were at lower risk of coronary heart disease, than men with a low leisure time physical activity, which was not seen in women [38].

Different measurements and definitions of physical activity complicates comparison between studies. Most previous studies on CVD focused on leisure time physical activity [34, 37, 39, 40], while few studies assessed total physical activity. Our study also differs from others by its detailed assessment of duration and intensity of total physical activity, summing up to 24 h/day. In a recent cohort study of men and women combined, the risk of MI was 29% lower among individuals with high total physical activity, measured as the total time spent in occupational, household, transport, and leisure time physical activity, as well as sitting time per week [41]. This finding is comparable with our 22% decreased risk among women. No inverse association was found between total physical activity and stroke, which also is consistent with our findings. The subjects were followed for a shorter period than in our study with a mean of 6.9 years, but they included 130,843 subjects from 17 different countries [41].

It might be more difficult to recall all activities for 24 h on an ordinary day, compared to recalling how much time per week that is spent on leisure time physical activity as exercise and sports. Our findings suggest, however, that the effect of different types of physical activity differ between men and women. Different types of physical activity could have different physiological effects, for example on sex hormones such as estrogen, which is known to have an effect on both vascular endothelium and triglyceride profile [42]. Environmental and social differences between men and women may lead to differences in the time and intensity spent on leisure time physical activity and other activities summing up to total physical activity. In our study, women tended to spend more time performing household chores, such as ironing and doing the dishes, while men spent more time in more strenuous housework, like mowing the lawn or shoveling snow. This could partly explain the differences seen in our study. In addition, it is more common that men have occupations with more physically strenuous work tasks. Previous studies have found high occupational physical activity to be associated with a slightly increased risk of CVD [13]. This may explain why we did not find an inverse association between total physical activity (which could be predominantly work dominated) and MI and stroke in men. The fact that occupational physical activity repeatedly has been shown to not lead to the same cardiovascular benefits as leisure time activity, constitutes the so called “physical activity paradox”. It has been hypothesized that occupational activity may be of too low intensity or too long duration to improve cardiovascular health; that heavy lifting and static positions increase blood pressure, and that poor worker control and lack of recovery time could be underlying mechanisms [43].

Our study has several limitations. All information was self-reported, as in other epidemiological studies. Further, information was only assessed at baseline, and participants may have changed their physical activity habits during the follow-up period. Self-reported physical activity has been shown to often be overestimated, while sedentary or light activity often are underestimated, or misclassified as moderate [14]. However, the potential misclassification of physical activity due to self-report is most likely equal among individuals with and without a subsequent MI and stroke. Such non-differential misclassification usually bias risk estimates towards a null effect, resulting in underestimated associations.

The total physical activity variable included sleep and sedentary activities whose effect to cardiovascular endpoints can differ from each other and compared to physical activities. This limitation might have effected to the associations of total PA and outcome measures reported in this study.

Our study includes a larger number of women compared to men, which may be a limitation since the prevalence of CVD differs between genders. The age-specific rates of CVD are higher in men than women in most age groups. Nevertheless, the lifetime risk of CVD is similar for both sexes, since prevalence in women increases later in life compared to men [44, 45]. At baseline, participants were on average 50 years old, and with a mean follow-up period of 17.9 years, the lack of associations between leisure time physical activity and MI and stroke in women may partly be explained by fewer cases where a longer follow-up time would be needed. Moreover, no association was found regarding stroke in women, which often occurs at older age than MI [45, 46].

Strengths of our study include the prospective design with a long and complete follow-up. Participation in the study was voluntary which might entail selection of individuals who are motivated to fill out the 36-page questionnaire carefully and correctly and the proportion of missing data is notably low. This enabled us to adjust for a number of potential confounders although we did not consider dietary factors. Also, the validity of the diagnosis for MI and stroke in the registers is high [25]. The large sample size including both sexes is a further strength, allowing us to study the associations in men and women separately. Furthermore, we used a validated questionnaire to assess detailed information about total physical activity.

## Conclusions

In conclusion, the results of this large prospective study suggest that total physical activity reduce the risk of MI in women, while participation in leisure time physical activity decrease the risk of MI and stroke in men. The differences between the sexes may be important in order to motivate and enhance the types of physical activity that contribute the most to a lowering of the risk of MI and stroke in the population. Future recommendations for physical activity in prevention of MI and stroke may want to consider these gender differences.

## Supplementary Information

**Additional file 1:Supplementary Figure 1. **A directed acyclic graph (DAG).

**Additional file 2. **Cubic spline models. LPA = Leisure time physical activity,TPA = Total physical activity.

**Additional file 3:Supplementary Table 1.** Distribution of METh per physical activity category during weekday presented for women and men in the Swedish National March Cohort.

## Data Availability

The datasets analysed during the current study are not publicly available due to ethical restrictions, but are available from the corresponding author on reasonable request.
