# Supplementary figures and images for: Association between total and leisure time physical activity and risk of myocardial infarction and stroke – a Swedish cohort study
Source: BMC Public Health. 2022 Mar 18;22:532. doi: 10.1186/s12889-022-12923-5 (PMC8932168; doi:10.1186/s12889-022-12923-5)

Supplementary figure 1


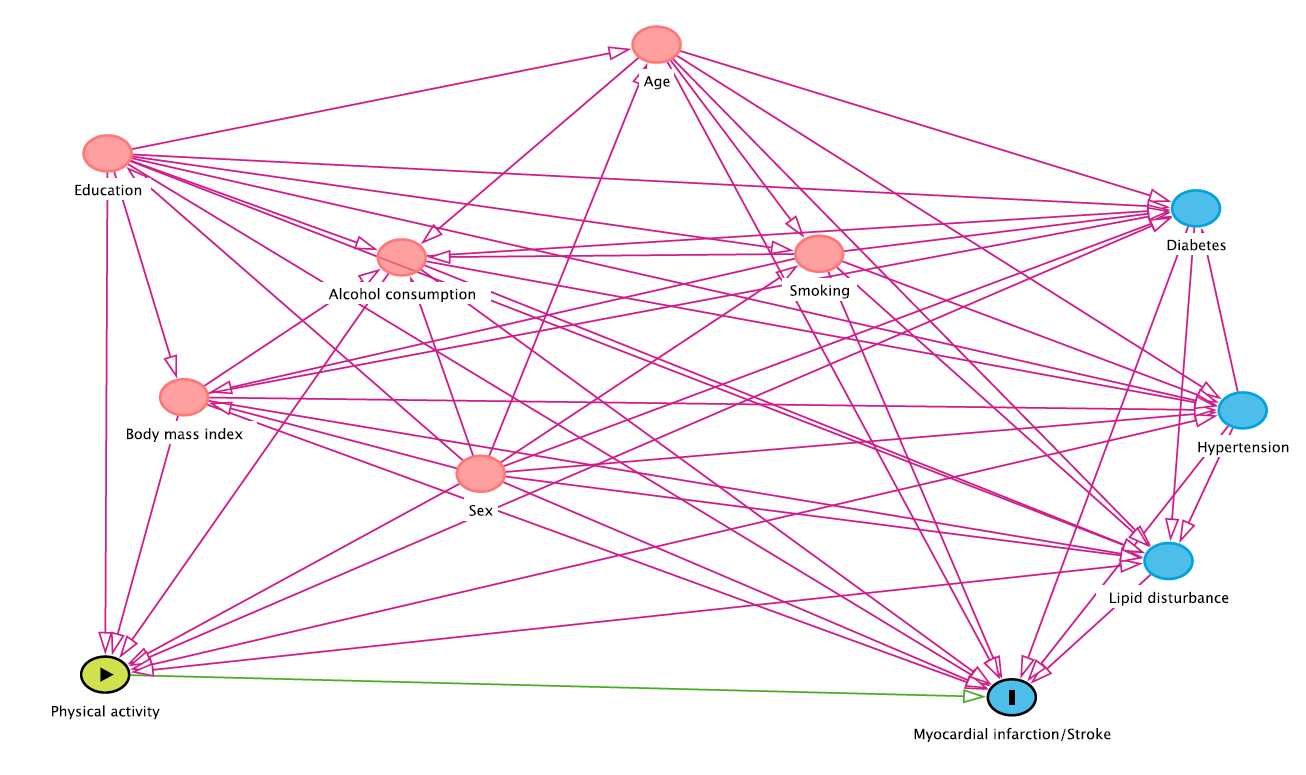

Supplement: Supplementary file 1 — Additional file 1:Supplementary Figure 1. A directed acyclic graph (DAG). [file 12889_2022_12923_MOESM1_ESM.docx]

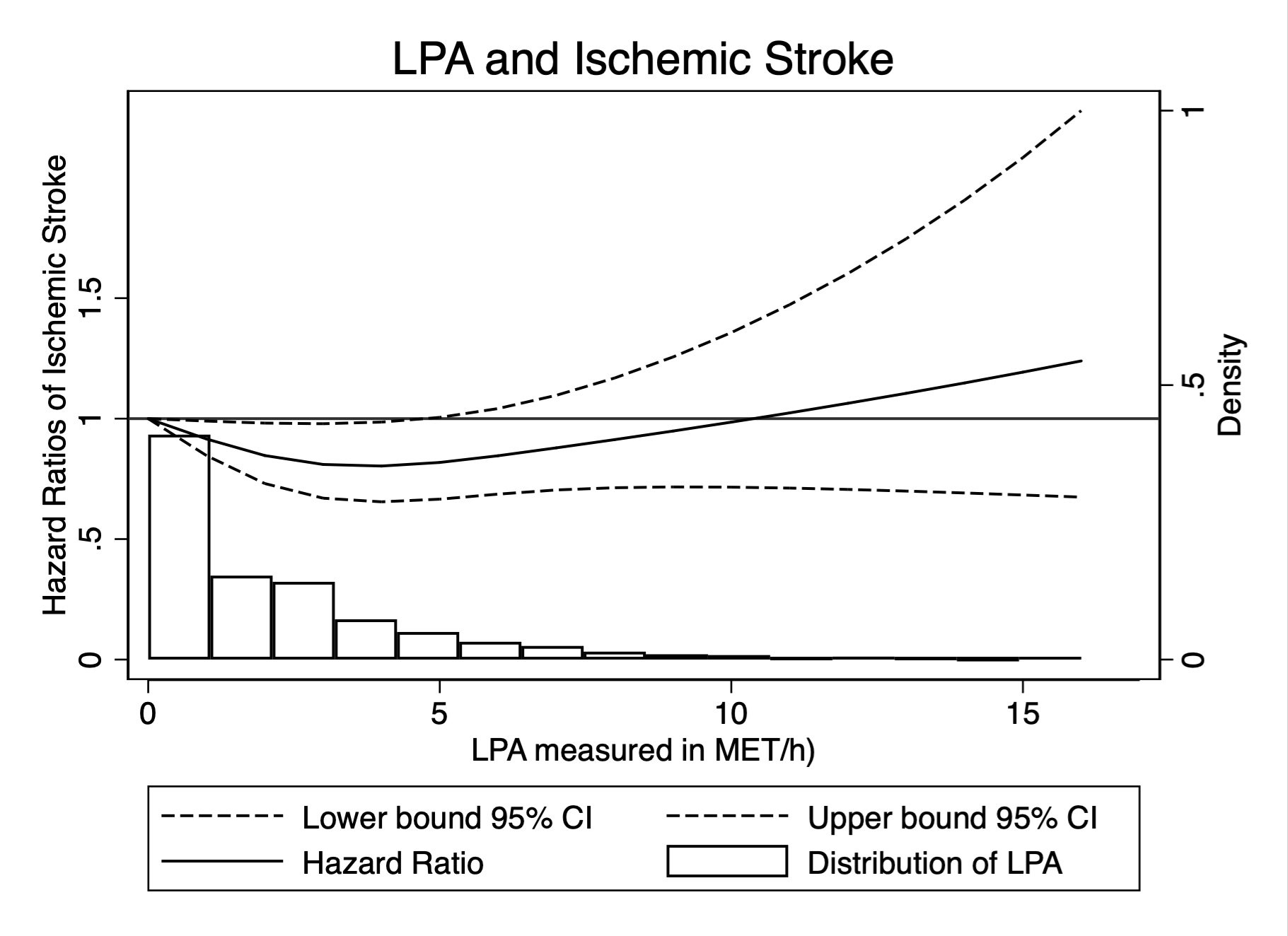

Supplement: Supplementary file 2 — Additional file 2. Cubic spline models. LPA = Leisure time physical activity,TPA = Total physical activity. [file 12889_2022_12923_MOESM2_ESM.zip › 1-2f. Supp_Figure-LPAandIschStroke-threek.tif]

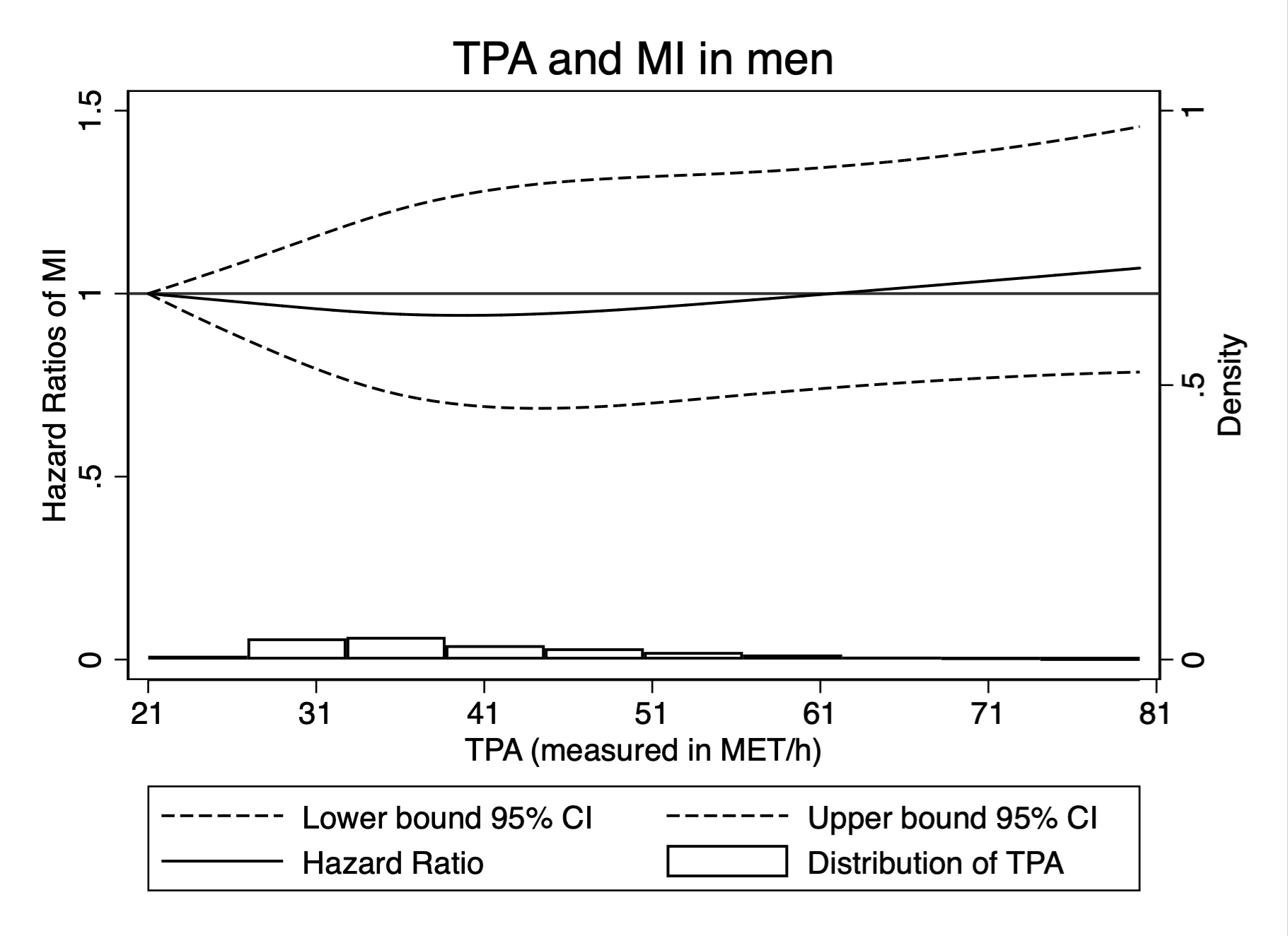

Supplement: Supplementary file 2 — Additional file 2. Cubic spline models. LPA = Leisure time physical activity,TPA = Total physical activity. [file 12889_2022_12923_MOESM2_ESM.zip › 1-2i. Supp_Figure-TPAandMImen-threek.tif]

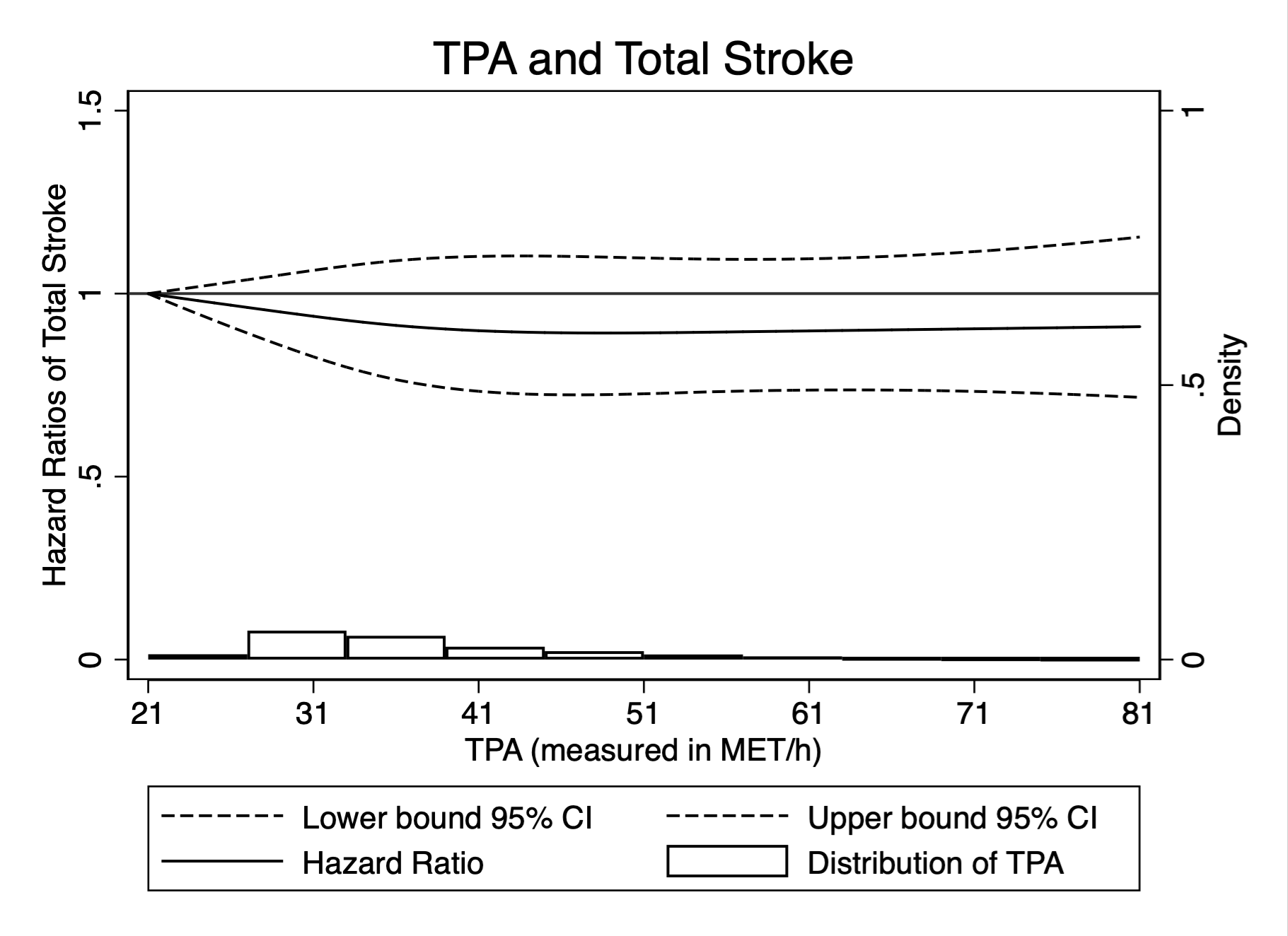

Supplement: Supplementary file 2 — Additional file 2. Cubic spline models. LPA = Leisure time physical activity,TPA = Total physical activity. [file 12889_2022_12923_MOESM2_ESM.zip › 1-2k. Supp_Figure-TPAandTotalStroke-threek.tif]

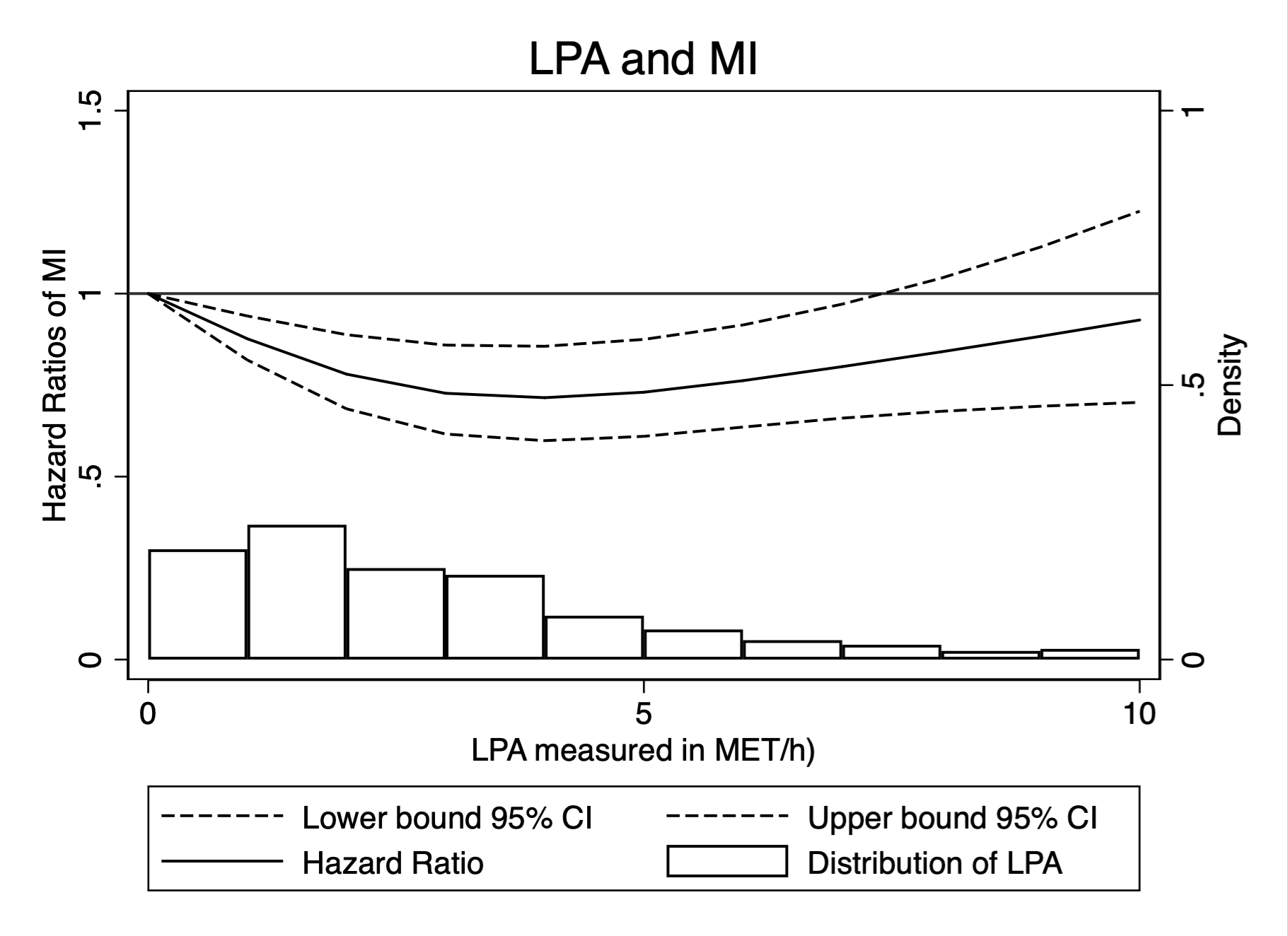

Supplement: Supplementary file 2 — Additional file 2. Cubic spline models. LPA = Leisure time physical activity,TPA = Total physical activity. [file 12889_2022_12923_MOESM2_ESM.zip › 2a. Supp_Figure-LPAandMI-threek.tif]

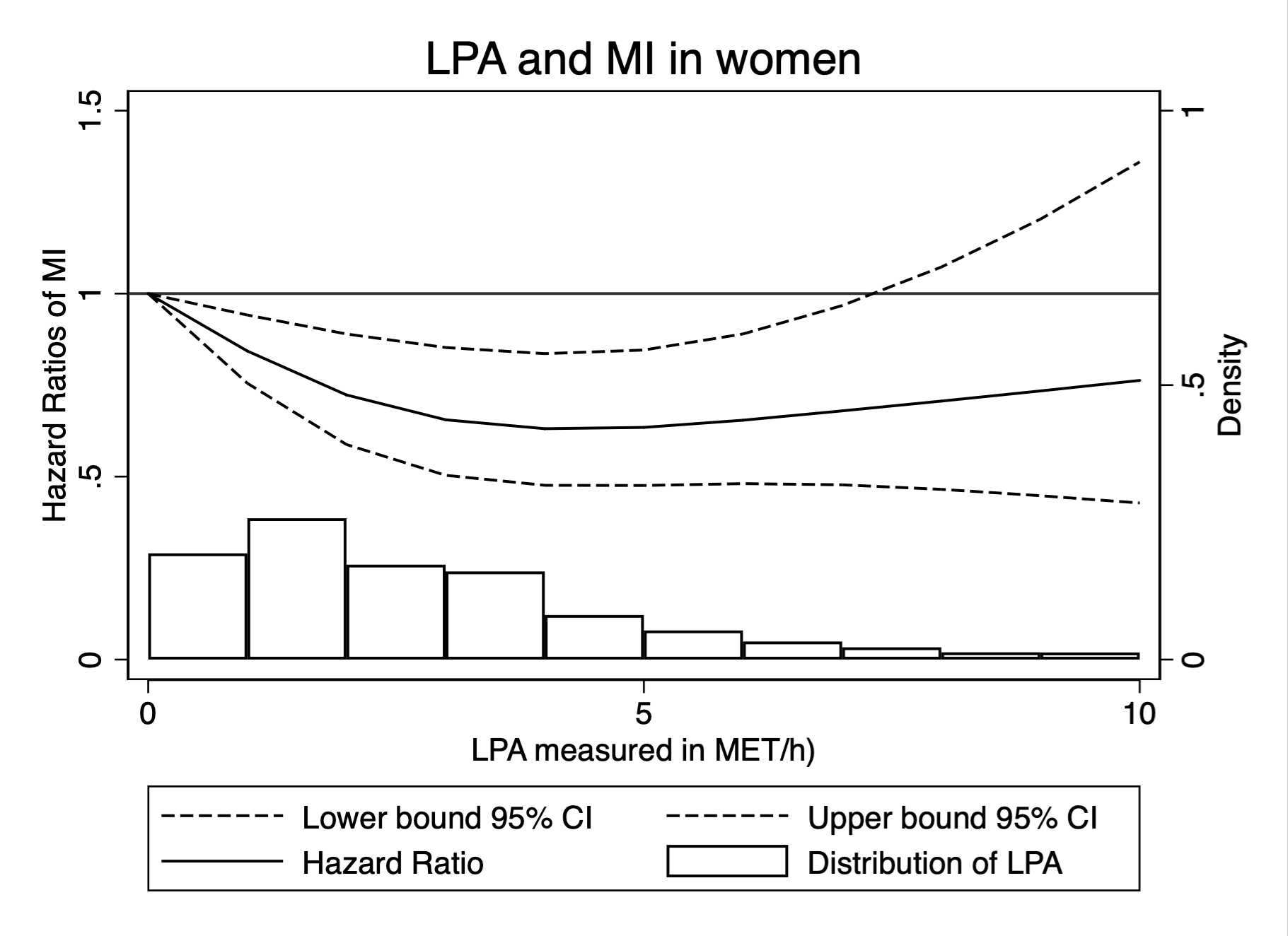

Supplement: Supplementary file 2 — Additional file 2. Cubic spline models. LPA = Leisure time physical activity,TPA = Total physical activity. [file 12889_2022_12923_MOESM2_ESM.zip › 2b. Supp_Figure-LPAandMIinWomen-threek.tif]

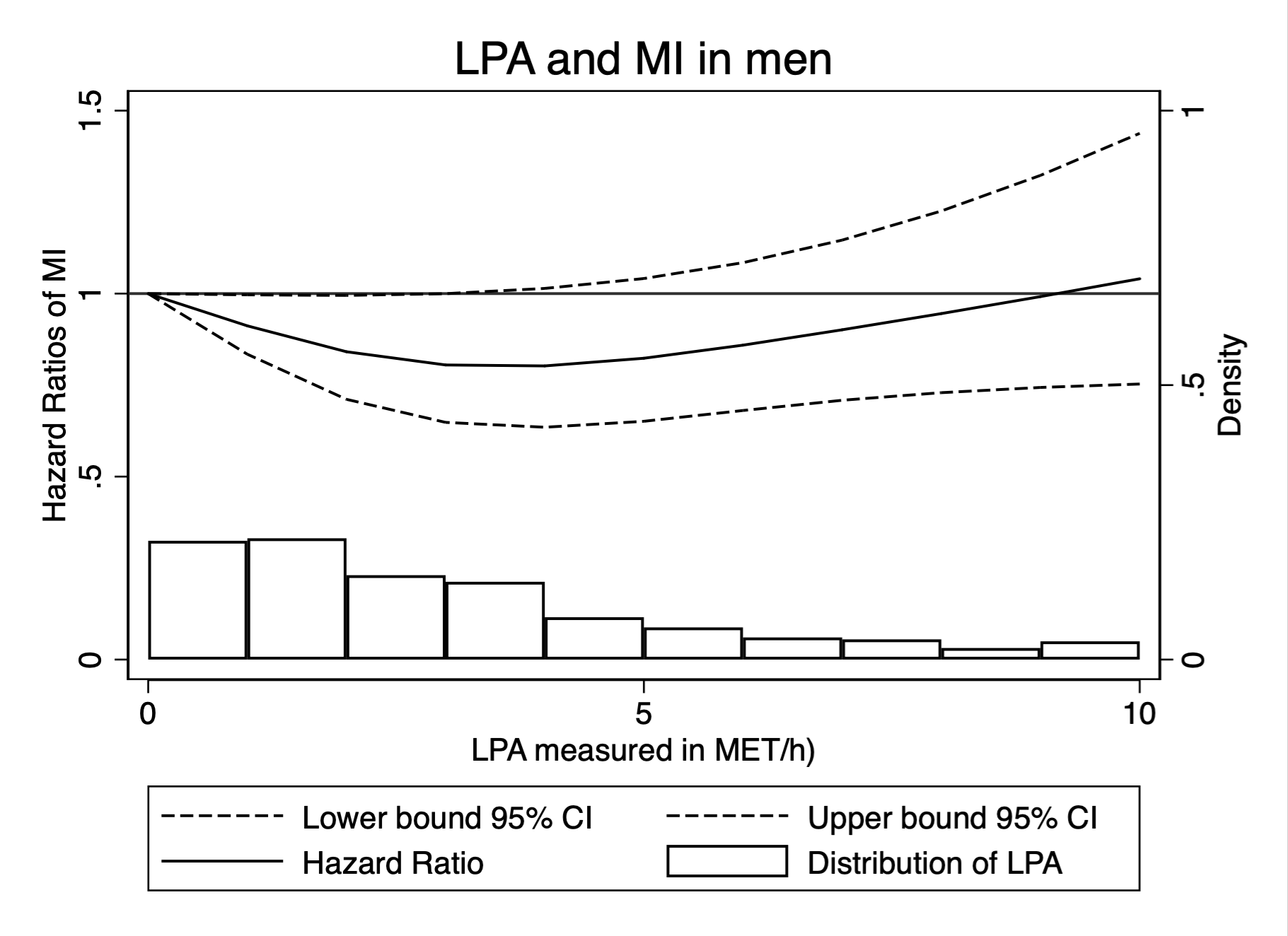

Supplement: Supplementary file 2 — Additional file 2. Cubic spline models. LPA = Leisure time physical activity,TPA = Total physical activity. [file 12889_2022_12923_MOESM2_ESM.zip › 2c. Supp_Figure-LPAandMIinMen-threek.tif]

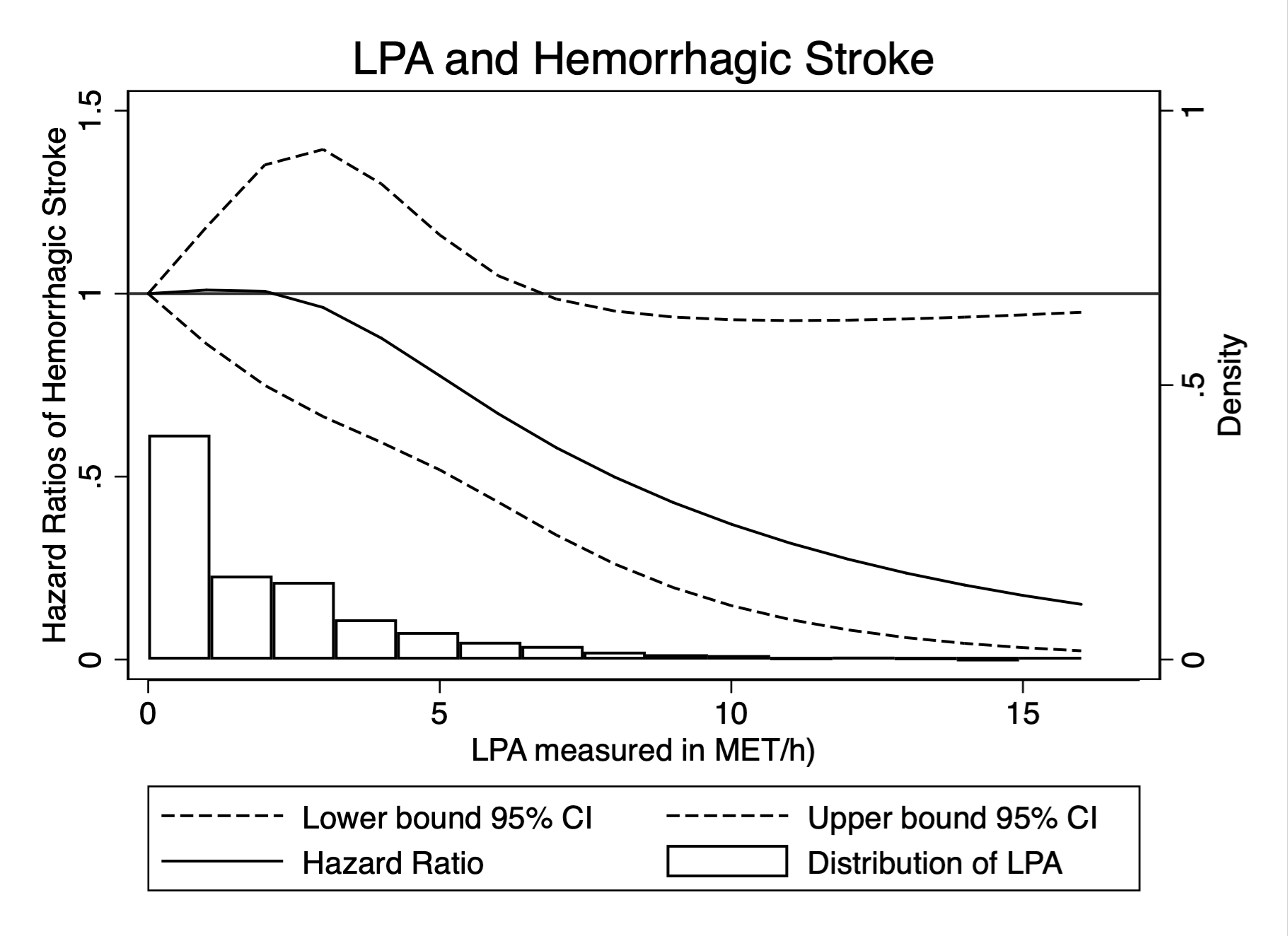

Supplement: Supplementary file 2 — Additional file 2. Cubic spline models. LPA = Leisure time physical activity,TPA = Total physical activity. [file 12889_2022_12923_MOESM2_ESM.zip › 2d. Supp_Figure-LPAandHemStroke-threek.tif]

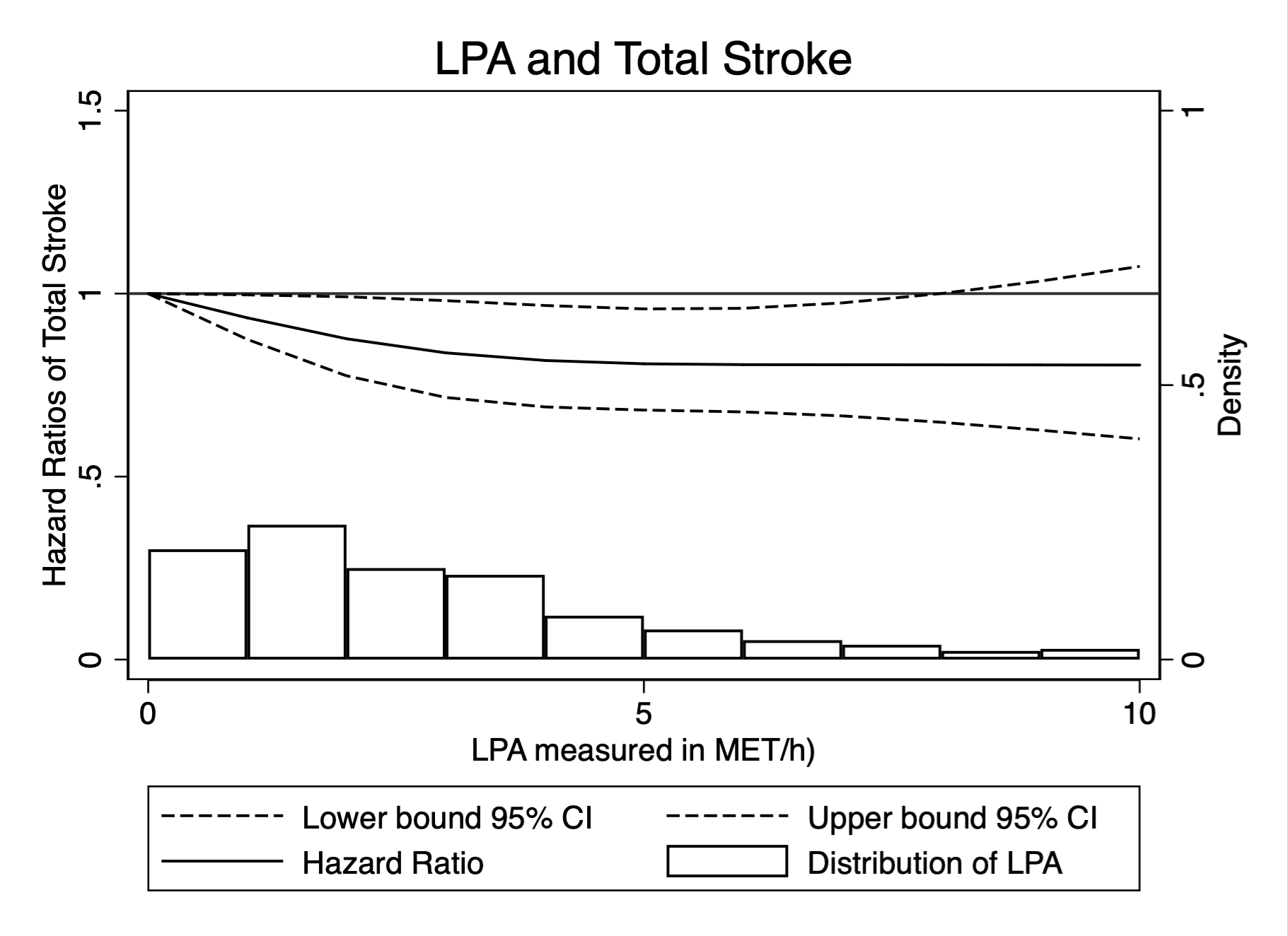

Supplement: Supplementary file 2 — Additional file 2. Cubic spline models. LPA = Leisure time physical activity,TPA = Total physical activity. [file 12889_2022_12923_MOESM2_ESM.zip › 2e. Supp_Figure-LPAandTotalStroke-threek.tif]

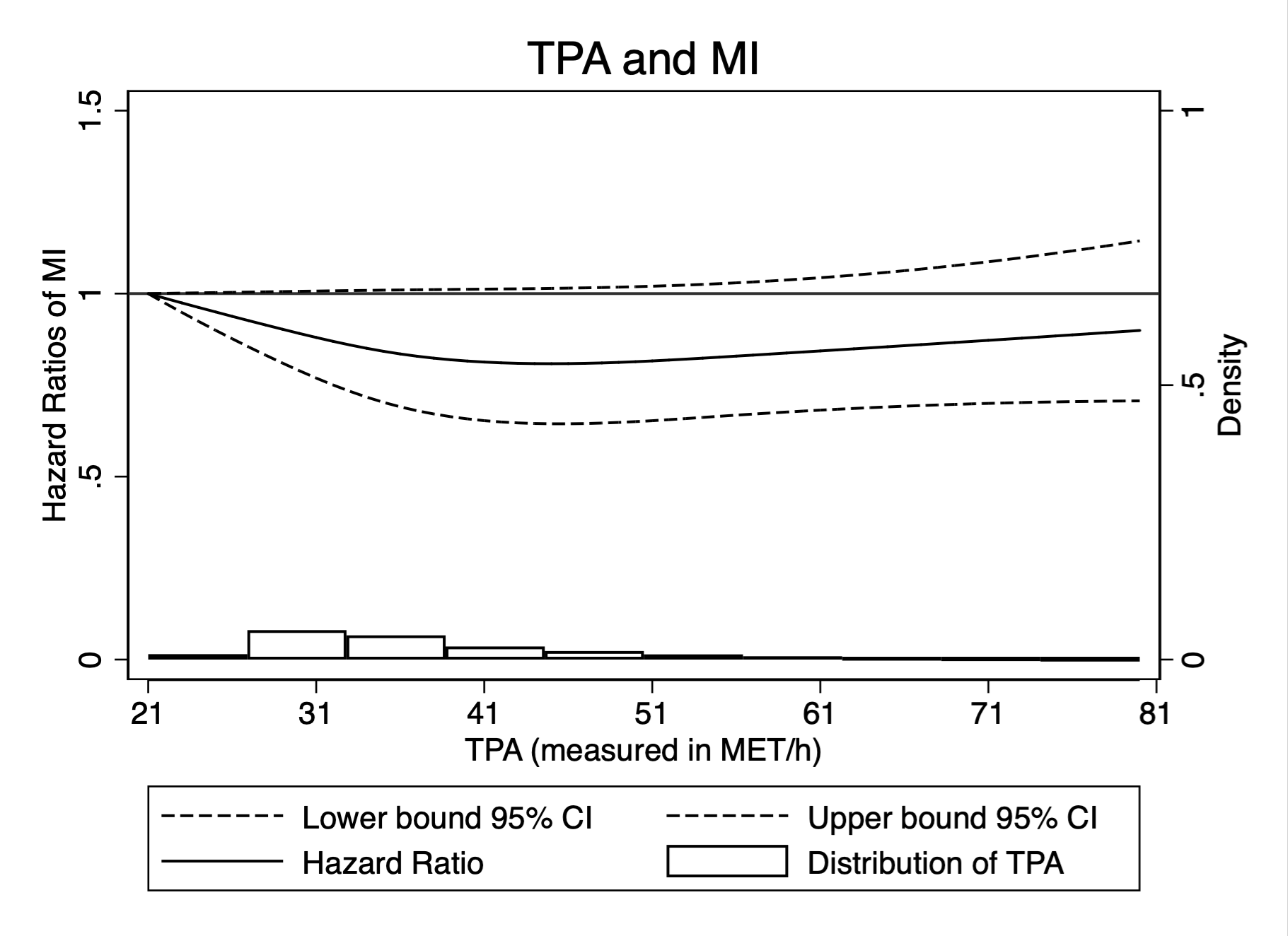

Supplement: Supplementary file 2 — Additional file 2. Cubic spline models. LPA = Leisure time physical activity,TPA = Total physical activity. [file 12889_2022_12923_MOESM2_ESM.zip › 2g. Supp_Figure-TPAandMI-threek.tif]

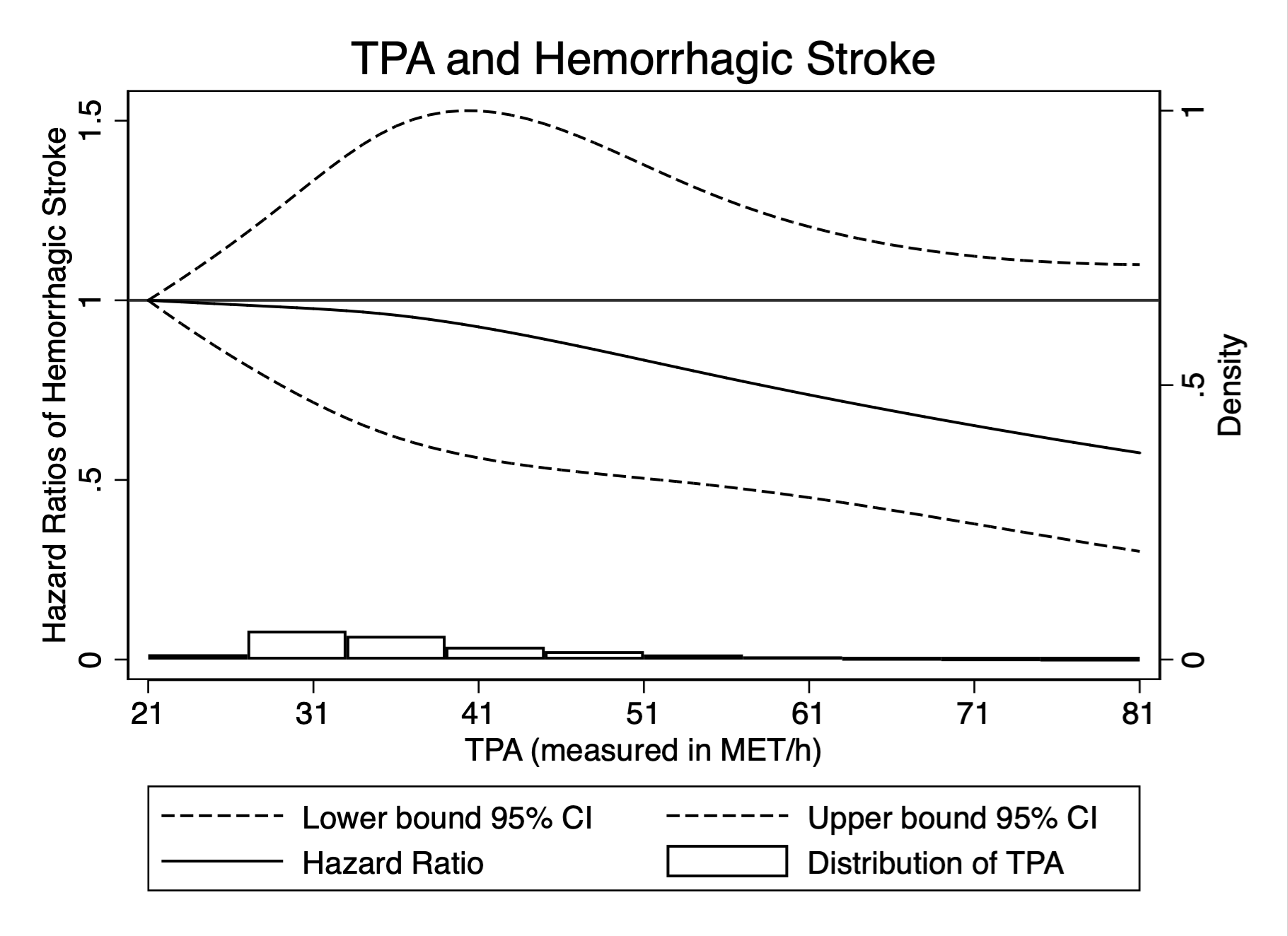

Supplement: Supplementary file 2 — Additional file 2. Cubic spline models. LPA = Leisure time physical activity,TPA = Total physical activity. [file 12889_2022_12923_MOESM2_ESM.zip › 2j. Supp_Figure-TPAandHemStroke-threek.tif]

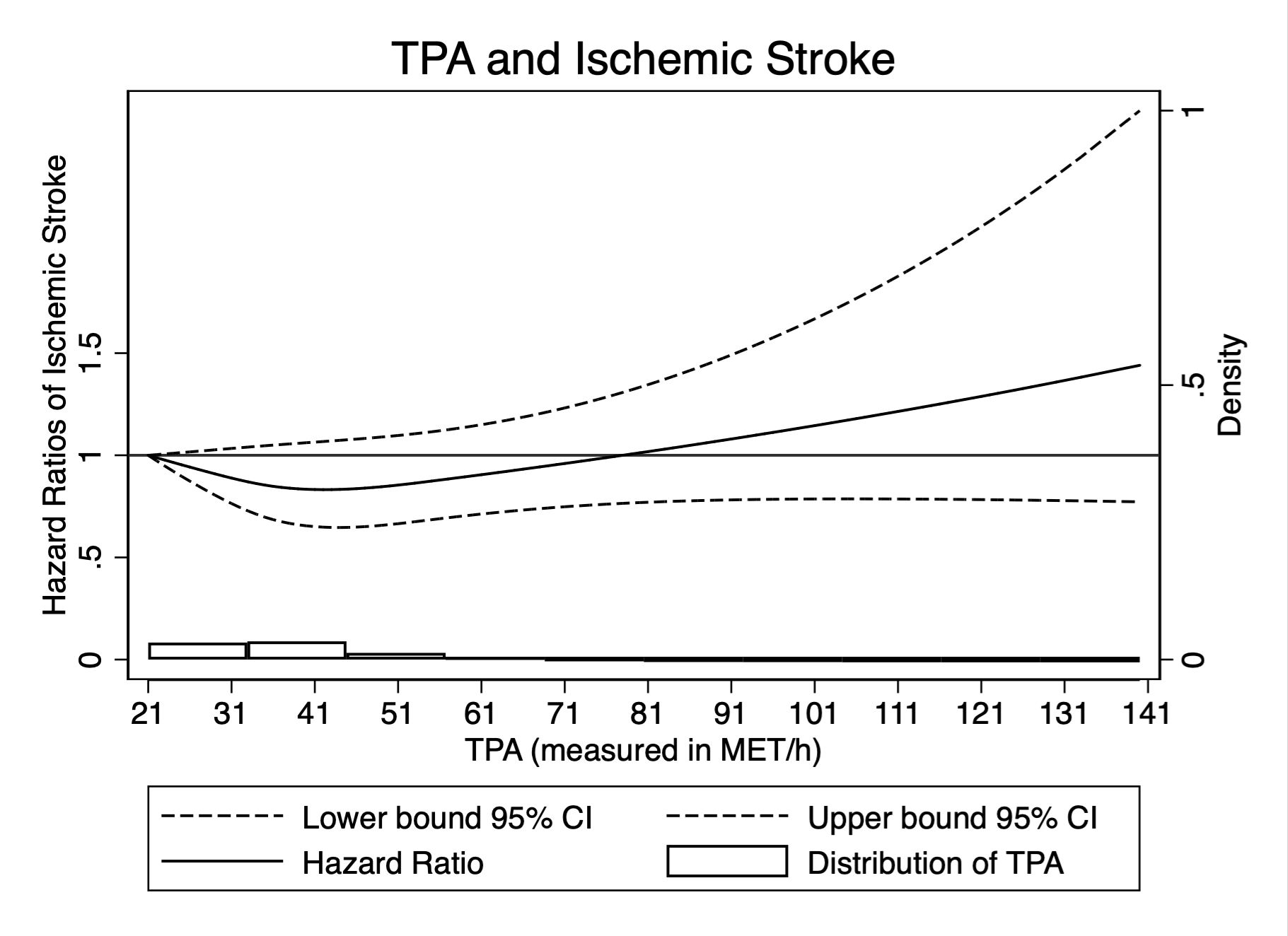

Supplement: Supplementary file 2 — Additional file 2. Cubic spline models. LPA = Leisure time physical activity,TPA = Total physical activity. [file 12889_2022_12923_MOESM2_ESM.zip › 2l. Supp_Figure-TPAandIschStroke-threek.tif]
