# Supplementary material for: Association between total and leisure time physical activity and risk of myocardial infarction and stroke – a Swedish cohort study
Source: BMC Public Health. 2022 Mar 18;22:532. doi: 10.1186/s12889-022-12923-5 (PMC8932168; doi:10.1186/s12889-022-12923-5)
Supplement: Supplementary file 3 — Additional file 3:Supplementary Table 1. Distribution of METh per physical activity category during weekday presented for women and men in the Swedish National March Cohort. [file 12889_2022_12923_MOESM3_ESM.docx]

| **Supplementary Table 1.** Distribution of METh per physical activity category during weekday presented for women and men in the Swedish National March Cohort. | | | | | | | | | |
| --- | --- | --- | --- | --- | --- | --- | --- | --- | --- |
| Physical activity during weekday | Sleep, rest | Sit in the bathtub, sit and listen on music, watch TV | Office work, knit,  sew, sit in a meeting | Make the bed, ironing,  washing up by hand | Bowling, drive bus/tractor,  workshop, fix the car,  dance walz/foxtrot | Quick walk, horse ride,  sweep the pavement | Paint the house, carry and  staple wood, ski/slalom | Roadwork, cut the lawn  with manual lawn mower,  shovel snow | Activities  more strenuous than the level before |
| MET per physical activity category | 0.9 | 1.0 | 1.5 | 2.0 | 3.0 | 4.0 | 5.0 | 6.0 | 8.0 |
| ***Female*** |  |  |  |  |  |  |  |  |  |
| Number | 22,226 | 21,925 | 21,771 | 22,112 | 20,552 | 21,484 | 20,669 | 20,766 | 19,861 |
| METh, Mean (SD) | 11.0 (2.8) | 2.9 (1.9) | 6.6 (5.8) | 3.6 (3.7) | 2.2 (4.6) | 4.1 (5.0) | 2.5 (4.8) | 2.7 (4.9) | 3.2 (8.3) |
| ***Male*** |  |  |  |  |  |  |  |  |  |
| Number | 11,232 | 11,075 | 10,889 | 10,892 | 10,673 | 10,837 | 10,661 | 10,794 | 10,185 |
| METh, Mean (SD) | 10.6 (2.8) | 3.0 (2.0) | 6.0 (6.2) | 1.4 (1.9) | 4.4 (7.5) | 3.8 (4.6) | 4.3 (6.5) | 5.0 (7.5) | 6.4 (12.6) |
|  | | | | | | | | | |
